# Assessing the reach and effectiveness of mHealth: evidence from a reproductive health program for adolescent girls in Ghana

**DOI:** 10.1186/s12889-017-4939-7

**Published:** 2017-12-20

**Authors:** Slawa Rokicki, Günther Fink

**Affiliations:** 1 Harvard Interfaculty Initiative in Health Policy, Harvard University, Cambridge, MA USA; 2 UCD Geary Institute for Public Policy, University College Dublin, Dublin, Dublin 4 Ireland; 3 Swiss Tropical and Public Health Institute and University of Basel, Basel, Switzerland

**Keywords:** Mobile health, Text messaging, SMS, Adolescent health, Sexual and reproductive health, Health promotion

## Abstract

**Background:**

While mobile health (mHealth) programs are increasingly used to provide health information and deliver interventions, little is known regarding the relative reach and effectiveness of these programs across sociodemographic characteristics. We use data from a recent trial of a text-messaging intervention on adolescent sexual and reproductive health (SRH) to assess the degree to which mHealth programs reach target adolescent subpopulations who may be at higher risk of poor SRH outcomes.

**Methods:**

The study was conducted among girls aged 14–24 in 22 secondary schools in Accra, Ghana. The mHealth intervention was an interactive mobile phone quiz in which participants could win phone credit for texting correct answers to SRH questions. We use detailed data on individuals’ level of engagement with the program, SRH knowledge scores, and self-reported pregnancy collected as part of the original trial to assess the extent to which engagement and program impact vary across parental education, sexual experience, SRH knowledge deficit, and parental support.

**Results:**

Eighty-one percent of participants engaged with the mHealth program, with no evidence that the program disproportionally reached better-off groups. The program was effective at increasing knowledge of SRH across all strata. Higher levels of engagement were associated with higher knowledge scores up to year later. There was no significant impact of the program on self-reported pregnancy within subgroups.

**Conclusion:**

mHealth programs for adolescents have the potential to engage and increase SRH knowledge of adolescent girls across sociodemographic strata, including those who may be at higher risk of poor SRH outcomes.

**Trial registration:**

ClinicalTrials.gov NCT02031575. Registered 07 Jan 2014.

## Background

With the rapid expansion of mobile phone ownership in low- and middle-income countries (LMICs) over the past decade, the field of mobile health (mHealth) has emerged as a novel and potentially cost-effective way to increase access to health information and improve health knowledge as well as health outcomes [1–4].

mHealth programs have been particularly popular in the area of adolescent sexual and reproductive health (SRH), where knowledge gaps remain large [5–11]. A recent review of evidence and progress over the past 20 years in adolescent SRH highlights the persistent lack of access to SRH information and services, as well as the large knowledge gaps with respect to use of contraception [12].

A growing body of evidence highlights early sexual debut and one-parent households as key risk factors for adolescent pregnancy and childbearing, and knowledge about contraception as a key protective factor against these outcomes [13]. Structural influences such as low parental socioeconomic status, low parental education and lack of parental support with regards to SRH have also been associated with adolescent risky sexual behaviour and inconsistent contraceptive use in the literature [13–15].

Recent evidence suggests that mHealth programs can increase average health knowledge and that these programs are generally well-received by youth [5–7]. However, very little is known regarding the reach and effectiveness of these programs in populations with key risk factors [16]. While mobile phone ownership and usage has greatly expanded among young people in many LMIC settings [17], adolescents from disadvantaged groups generally have reduced rates of phone ownership or may only have limited access to a shared household phone [18–21]. In addition, youth from these key populations may face other barriers pertinent to mHealth such as decreased technological literacy, inferior network coverage, and lower linguistic competency [2, 3].

In this study, we analyse detailed data collected as part of a randomized trial of a text-messaging intervention conducted among secondary school students in Accra, Ghana. As part of the trial, adolescents were invited to participate in an interactive mobile phone program aimed to increase knowledge of SRH. We seek to answer the following research questions: (1) how effective was the program in engaging adolescents from key target populations including adolescents with low parental education, early sexual debut, low parental support, and low knowledge of SRH; (2) how did the impact of the program on SRH knowledge vary with level of engagement; and (3) how successful was the program at improving SRH knowledge and reducing incidence of pregnancy in key target populations?

## Methods

### Study setting

The study was conducted in the urban area of Accra, Ghana [1]. In a survey from 2015 in Ghana, 31% of respondents aged 14–18 years and 71% of respondents aged 19–25 years owned a mobile phone, while 77% of those aged 14–18 and 91% of those aged 19–25 used a mobile phone in the past 4 weeks [20].

Despite enacting a National Adolescent Reproductive Health Policy in 1996, the SRH of Ghanaian adolescents remains a significant challenge. According to the 2014 Demographic and Health Survey, by age 19, 36% of women have given birth or are pregnant [22]. Among adolescent mothers under 20 years of age, 58% had not planned on having the pregnancy at that time. Use of contraception among adolescents is low: among sexually active unmarried adolescents aged 15–19, less than a third use a modern method of contraception [22].

Though adolescents are generally aware of HIV and pregnancy risks, SRH knowledge tends to be limited and mostly superficial. Results of a 2004 national survey of Ghanaian adolescents found that while more than 95% of girls aged 15–19 knew of at least one method of modern contraception, only 67% were aware that a woman is more likely to get pregnant on certain days than others. Only 28% knew both previous items and were able to reject two common misconceptions (that a woman cannot get pregnant the first time she has sex or if she has sex standing up) [23]. Moreover, widespread misconceptions about the correct way to use contraception and fears about long-term side effects on future fertility add to the barriers to modern contraception use among adolescents [24–26].

### Procedures

We used secondary data from a recent randomized trial that provided SRH information by text message (also known as short message service, or SMS) to adolescent girls in Ghana. The original trial, which is described in further detail in Rokicki et al. (2016), found relatively large and persistent increases in SRH knowledge among adolescents participating in the interactive mHealth program [5].

The sampling frame for the study was provided by the 2012–2013 Ghana Education Service Register of Secondary Schools in Greater Accra. We randomized 38 schools to the interactive mHealth intervention (*n* = 12), a simplified unidirectional messaging intervention (*n* = 12), and the control group (*n* = 14). After randomization, we found 3 schools to be ineligible and 1 refused to participate due to time constraints [5]. In this paper, we focus only on the interactive mHealth intervention arm and the control arm. The analytic sample consisted of 10 schools assigned to the intervention (*N* = 205) and 12 schools assigned to control (*N* = 293).

We blocked randomization by school category (a measure of quality designated by the Ghana Education Service) and by whether the school had a home economics class. We chose classes within schools based on maximizing the number of eligible girls; if a home economics class was offered in the school it was chosen because it was always a large class of nearly all female students. Home economics was not offered at all schools so stratifying ensured even distribution across arms.

The inclusion criteria for schools was senior high day schools in the Greater Accra region; boarding schools were excluded. Within schools, sampling was restricted to female students, aged 14–24 years, in one class in the second year of senior high school. Participants used their own mobile phones or could use a family member’s phone; no phones were provided.

Participants gave written consent; those aged younger than 18 years obtained parental consent. Institutional Review Board (IRB) approval was granted by Harvard University [#FWA00004837] as well as locally by the Ghana Health Service (GHS-ERC:05/09/13). The study design was registered on ClinicalTrials.gov (NCT02031575).

We visited schools at baseline, immediately after the intervention was completed (3-month follow-up), and 1 year later (15-month follow-up). At each visit, participants completed a self-administered questionnaire. Participants’ demographic information was completed at baseline. Reproductive health knowledge was assessed at each time-point. Information on sexual behavior and pregnancies was collected only at the 15-month follow-up.

### The mHealth platform

The mHealth platform was designed as an interactive mobile phone quiz game in which participants could win airtime (i.e. mobile phone credit that can be used for making calls or sending texts) for texting correct answers to SRH questions. To design the message content, we first conducted focus groups with adolescents to understand their most prevalent SRH concerns, followed by a round table discussion with the Health Promotion Unit at Ghana Health Service who approved appropriateness of topics and finalized wording.

For a period of 12 weeks, participants were sent one multiple-choice quiz question about SRH each week via text message to which they were invited to respond free of charge. These messages focused on pregnancy prevention and contained information on topics of reproductive anatomy, pregnancy, sexually transmitted infections (STIs), and contraception including male and female condoms, birth control pills, and emergency contraception. Upon responding, participants immediately received a confirmatory text message informing them whether they answered correctly, the correct answer, and additional information. Participants were sent up to 2 reminder messages if they did not respond; those who had not responded by the end of the week were sent a text message with the correct answer and the additional information at the end of the week. Participants in the same school were encouraged to discuss messages with each other. Participants were told that correct answers were rewarded: for every 2 correct responses, participants were sent 1 GHS (US$0.38) of airtime credit at the end of the week.

Over the same 12-week intervention period, the control group participants were sent one message each week with information about malaria. The content of all messages is shown in Table 4 in Appendix. Participants in the control group were interviewed at baseline, 3 months, and 15 months using the same procedures as participants in the intervention group. All messages were sent in English, the official language of instruction in all secondary schools in the country, using secure servers via Telerivet service; non-delivered messages were re-sent.

### Measures

To measure program engagement, we used two separate variables. First, we measured the total number of times the respondent replied to the weekly text-message quiz questions (maximum of 12), from data extracted from the mobile phone records. Second, we measured self-reported message exposure. At the 3-month follow up, respondents were asked “How often did you receive messages from [the program]” [More than once a week/ About once a week/ About once a month/ Less than once a month]. We created an indicator for having received messages at least once a week.

To evaluate reproductive health knowledge, participants completed a true-or-false test consisting of 24 items (see Table 5 in Appendix for details). Items on the test were adapted from the Guttmacher Institute’s 2009 National Survey of Reproductive and Contraceptive Knowledge for the setting of Ghana [27]. Knowledge scores were calculated as the percentage of items answered correctly; we then calculated knowledge z-scores by subtracting from each score the overall mean at baseline and dividing by the standard deviation.

In calculating knowledge scores, “don’t know” answers and missing values were treated as incorrect answers. The percentage missing for each item was low and is shown in Table 5 in Appendix. At the 15-month follow-up, data collection was done using tablet computers so there were no missing values in those scores. For the baseline and the 3-month follow-up, which were done on paper questionnaires, we re-calculated knowledge scores such that items with missing values were excluded from the calculation (that is, the knowledge score was calculated as the percentage correct of the total number of items answered). The correlation coefficients between treating missing values as incorrect and excluding missing values from the calculation was 0.92 at baseline and 0.99 at the first follow-up.

Self-reported pregnancy was assessed at the 15-month follow-up with the question “In the past year, have you been pregnant?” [Yes/ No/ I don’t want to answer].

Our explanatory variables were selected ex-ante on the basis of theory and prior evidence of risk and protective factors for adolescent SRH [13]. We identified these as: 1) adolescents from households of low socioeconomic status (defined as both parents completed only primary school or less), 2) adolescents who were sexually active at baseline, 3) adolescents who have a larger than average SRH knowledge deficit (earned a baseline knowledge z-score of less than 0), and 4) adolescents who have low parental support (do not strongly agree or agree a little bit with the statement “I feel comfortable talking to my parents about condoms and contraception” [Strongly agree, Agree a little bit, Neither agree nor disagree, Disagree a little bit, Strongly disagree]).

### Statistical analysis

Our analysis is divided in three parts. First, we evaluated characteristics associated with engagement with the interactive mHealth program. We used a Poisson regression model to examine the association between number of responses to the text-messsage quiz questions as our outcome and parental education, sexual experience, parental support, and SRH knowledge as explanatory variables. We also verified similarity of results to a negative binomial model to account for over-dispersion (Table 6 in Appendix). Additionally, we used logistic regressions to examine the associations of these characteristics with a binary variable for any response to the text-messages, as well as with a binary variable measuring participation as whether the participant self-reported to have received messages at least once a week during the course of the program.

Next, to evaluate the impact of engaging with the program on reproductive health knowledge, we used linear regression models of knowledge z-score at both 3 and 15 months as a function of level of engagement (number of responses) of the intervention group. We used quantile-quantile plots to assess normality of the residuals.

Finally, we assessed variation in program impact across target subgroups. We used linear regression models stratified by subgroup to estimate the impact of the intervention on knowledge z-score. We then used models with an interaction term for intervention group and subgroup indicator to test equality of the coefficients. For these interaction tests, marginal effects were derived from the interaction model, and then compared using linear hypothesis tests.

Due to the small cell sizes in subgroups, we used exact logistic regression models, again stratified by subgroup, to estimate the impact of the intervention on self-reported pregnancy in the past year.

Missing values and item response refusals were included in the analyses as separate categories; results for these categories are shown in Tables 6 and 7 in Appendix. We adjusted all models for blocking variables, which were category of school (higher quality public, lower quality public, and private) and an indicator for the presence of home economics class) and we clustered standard errors at the school level to correct for within-school correlation of outcomes [28, 29]. We used Stata v14 for all analyses [30].

## Results

Table 1 shows the baseline characteristics of the participants, which were similar for intervention and control groups. At baseline, 58% of participants were 17–18 years of age, while 18% were 16 and under and 24% were 19 and over. Almost 70% of participants were not sexually active at baseline while 13% were sexually active; another 13% refused to answer the question. 8% of participants reported that both parents had low education (primary or less), 23% reported that at least one parent had higher education, 49% reported that both parents had higher education, while 20% did not know. About a third of participants (32%) reported that they had high parental support, while the rest reported low parental support.

**Table 1:** Baseline risk profile and socioeconomic characteristics (n (%))

	Intervention, *N* = 205	Control, *N* = 293	Total, *N* = 498
Age			
< =16 years	44 (21)	46 (16)	90 (18)
17–18 years	116 (57)	172 (59)	288 (58)
> =19	45 (22)	75 (26)	120 (24)
Sexually active			
No	147 (72)	198 (68)	345 (69)
Yes	26 (13)	39 (13)	65 (13)
Refused to answer	24 (12)	39 (13)	63 (13)
Missing	8 (4)	17 (6)	25 (5)
Knowledge score			
No knowledge deficit	119 (58)	135 (46)	254 (51)
Large knowledge deficit	86 (42)	158 (54)	244 (49)
Parental education			
Both parents low education	12 (6)	28 (10)	40 (8)
One parent high education	49 (24)	66 (23)	115 (23)
Both parents high education	108 (53)	134 (46)	242 (49)
Don’t know/Missing	36 (18)	65 (22)	101 (20)
Parental support			
Low	137 (67)	202 (69)	339 (68)
High	67 (33)	91 (31)	158 (32)
Missing	1 (0)	0 (0)	1 (0)
Religion			
Muslim	24 (12)	52 (18)	76 (15)
Catholic	18 (9)	21 (7)	39 (8)
Protestant	54 (26)	61 (21)	115 (23)
Charismatic/Other	105 (51)	154 (53)	259 (52)
Missing	4 (2)	5 (2)	9 (2)
Ethnicity			
Akan/Fanti	70 (34)	112 (38)	182 (37)
Ga/Ewe/Other	132 (64)	169 (58)	301 (60)
Missing	3 (1)	12 (4)	15 (3)

Notes: Protestant includes Methodist and Presbyterian. Charismatic/Other includes Spiritual and Pentecostal. Low education defined as completed primary school or less

### Differences in program engagement by target subgroups

Table 2 shows the results of the Poisson and logistic regression models investigating characteristics associated with engagement with the mHealth program. Overall, responsiveness to the program was high: 81% responded via text message to at least one quiz question; the average number of responses was 8. The histogram of responses is shown in Figure 3 in Appendix. Adjusting for other covariates, those who reported that both parents had low education had a significantly higher rate of responding to messages (Incidence Rate Ratio (IRR) 1.22, 95% CI 1.03–1.46), but were not more likely to report receiving messages. We found no difference in program engagement by baseline sexual activity, knowledge, or parental support.

**Table 2:** Characteristics associated with program engagement as measured by number of responses, response to any messages, and self-reported message receipt (intervention group only)

	Number of responses	Responded to any message	Self-reported received messages at least once a week
	IRR (95% CI)	OR (95% CI)	OR (95% CI)
Sexually active at baseline (Ref: Not active)	1.08 (0.86–1.37)	1.60 (0.49–5.22)	1.99 (0.26–15.2)
Knowledge deficit (Ref: No knowledge deficit)	0.92 (0.78–1.09)	0.95 (0.41–2.21)	0.58 (0.21–1.55)
Parental education (Ref: Both parents high education)			
Both parents low education	1.22 (1.03–1.46)	1.98 (0.28–14.1)	0.79 (0.18–3.41)
One parent high education	0.93 (0.84–1.03)	0.83 (0.30–2.31)	1.13 (0.29–4.50)
Low parental support (Ref: High parental support)	1.04 (0.85–1.28)	1.71 (0.70–4.19)	1.60 (0.69–3.67)
Observations	204	204	191
Mean (SD)	8.0 (4.8)	0.81 (0.39)	0.83 (0.37)
Median (IQR)	11 (4–12)		

*Notes*: Results from 3 regression models of each outcome on full set of explanatory variables. Separate categories for “Refused to answer” and “Don’t know/Missing” were included in the model (see Table 6 in Appendix for these results). Models adjusted for blocking variables (category of school and presence of home economics class). *Ref* reference category, *IRR* incidence rate ratios, obtained from a Poisson regression, *OR* Odds ratios, obtained from logistic regression, *msgs* messages, *CI* confidence interval, *SD* standard deviation, *IQR* Interquartile range

### Association between engagement and knowledge

**Fig. 1:**
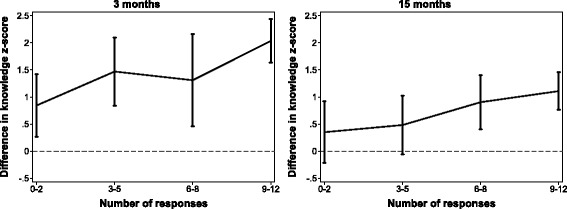
Average difference in knowledge z-score between intervention and control groups, at 3 months (left) and 15 months (right), as a function of number of responses to text message quiz questions (uses intervention and control groups, *N* = 498)

Figure 1 shows the difference in average knowledge z-score between intervention and control groups as a function of the intervention group's engagement with the program (as measured by the number of responses). Higher levels of engagement were associated with higher knowledge scores both at 3 months (linear slope estimate 0.11, 95%CI 0.08 to 0.14) and at 15 months (linear slope estimate 0.07, 95%CI 0.02 to 0.13).

### Difference in program impact on high risk groups

**Fig. 2:**
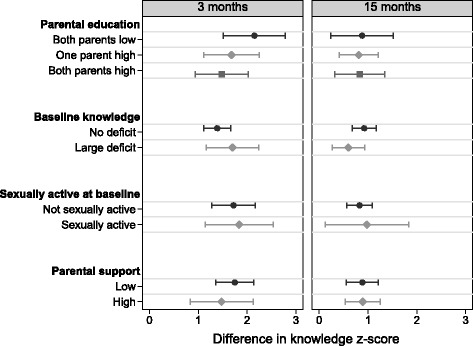
Difference in knowledge z-score for intervention group compared to the control group at 3 and 15 months, stratified by target subgroups (uses intervention and control groups, *N* = 498)

**Fig. 3:**
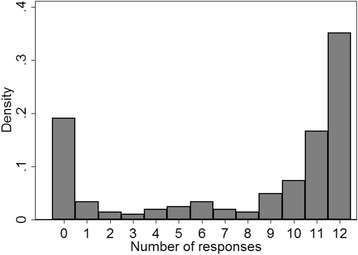
Distribution of number of responses

Figure 2 shows the difference in knowledge z-score for the intervention compared to the control, stratified by target subgroups. The mHealth program was effective at significantly increasing knowledge for every subgroup at both 3 and 15 months. We found no heterogeneity in the effect of the intervention across any subgroup population (Table 7 in Appendix).

Finally, Table 3 shows the impact of the mHealth program on self-reported pregnancy in the past year (reported at 15 months), stratified by target subgroups. Among those sexually active at baseline, 2 of 25 participants (8%) in the intervention group reported a pregnancy, while 6 of 39 participants (15.4%) in the control group reported a pregnancy. However, the odds ratio estimate was not significant and confidence intervals were wide due to the small cell sizes in these subgroups.

**Table 3:** Relative program impact on self-reported pregnancy in the past year for target subgroups (uses intervention and control groups, N = 498)

	Events/participants n/n (%)		Exact logistic results	
Subgroup	Control	Intervention	OR	95% CI
Baseline sexual activity				
Not sexually active	1/197 (0.5%)	2/144 (1.4%)	2.58	(0.12–163.3)
Sexually active	6/39 (15.4%)	2/25 (8%)	0.17	(0.003–1.92)
Refused	3/39 (7.7%)	2/24 (8%)	2.83	(0.21–29.01)
Parental education				
Both parents low	3/24 (12.5%)	2/12 (16.7%)	2.24	(0.14–28.32)
One parent high	3/64 (4.7%)	2/48 (4.2%)	1.03	(0.07–10.69)
Both parents high	4/123 (3.3%)	1/98 (1.0%)	0.14	(0.00–1.43)
Missing/Don’t know	0/65 (0%)	1/35 (2.9%)	–	–
Baseline knowledge				
No deficit	6/126 (4.8%)	5/113 (4.4%)	0.85	(0.18–3.67)
Large knowledge deficit	4/150 (2.7%)	1/80 (1.3%)	0.50	(0.01–6.29)
Parental connectedness				
Not connected	6/193 (3.1%)	5/131 (3.8%)	1.42	(0.31–6.10)
Connected	4/83 (4.8%)	1/61 (1.6%)	0.20	(0.003–2.47)

Notes: *OR* Odds ratio, *CI* confidence interval. Coefficients derived from exact logistic regression models stratified by subgroup. Models adjusted for blocking variables (category of school and presence of home economics class)

## Discussion

The results presented in this paper suggest that mHealth programs are not only an effective tool to increase SRH knowledge overall in the studied setting, but that these programs can also engage and increase SRH knowledge of adolescents from key target populations who are at higher risk of poor SRH outcomes, including adolescents with low parental education, adolescents with low SRH knowledge, adolescents with early sexual debut, and adolescents with low parental support.

We found that overall engagement in the program was high, and that participants in target groups were just as likely to interact with the program as their counterparts. In fact, participants whose parents had low levels of education had a higher rate of responding to messages than those whose parents had high levels of education.

Program engagement also appears to be important for knowledge absorption. We found that those who interacted more with the program had significantly greater knowledge of reproductive health in both the short-term (immediately after the program ended) and in the longer-term (a year later), compared to those who did not interact. Those who did not reply to messages still received the reproductive health information at the end of each week; however, knowledge improvements were minimal at 3 months and not significantly different from the control at 15 months. We cannot interpret the differences causally, as there may be confounding factors such as motivation that affect both desire to interact with the program and knowledge. However, the findings point to the potential importance of including features such as interaction and feedback in the design of mHealth programs for adolescents [3, 31].

We also found that the mHealth program was effective at increasing SRH knowledge across levels of parental education, SRH knowledge, sexual experience, and parental support, in both the short-term and long-term, a surprising finding considering the number of potential cultural and technological barriers that participants in target groups may encounter.

Finally, we found no significant impact of the mHealth program on self-reported pregnancy within subgroups. Although girls in the intervention group who were sexually active at baseline had fewer self-reported pregnancies than their counterparts in the control group, the difference was not significant and sample sizes were too small to draw strong conclusions. Providing information via mobile phone to the population that needs the information most – in this case, girls who are sexually active and who immediately benefit from information about contraception and pregnancy prevention – may be an effective way of reducing unintended pregnancy rates; however, the study analyzed here was not powered to directly address this question, and more research is needed to understand the effect of mHealth programs on unintended pregnancy in sexually active adolescent populations.

This analysis has a number of limitations. First, there may be measurement error in some of the outcomes studied. Sexual activity and pregnancy were self-reported and thus may be subject to recall bias and social desirability bias. The bias may be differential by intervention group if, for example, provision of sexual health information by mobile phone made participants more likely than those in the control group to respond accurately and honestly to SRH questions. It may also be differential by target subgroup; for example, those with stronger parental connectedness or more baseline SRH knowledge may also respond more honestly. However, neither the size nor the direction of such biases is obvious. In other studies, adolescents’ self-reported sexual behavior data has been reported to suffer from inconsistencies, recall error, and misunderstanding or confusion surrounding the survey question [32]. In future studies, biological markers of pregnancy would be ideal to fully understand the health impact of the program. Another data limitation of the study presented is that a large proportion of girls (20%) did not know the level of their parent’s education. It is also worth noting that participants were recruited from secondary school in an urban area and thus are not representative of the general population; heterogeneity in terms of socioeconomic and demographic characteristics in the general population may be much larger. In particular, girls from rural areas and girls who stopped schooling before secondary school may have less access to mobile phones or reduced literacy skills than the girls in our sample. As this study was a small scale pilot study, we did not include boys. In terms of interpreting impacts, the intervention was a multi-component program that included participant interaction with quiz questions, reminder messages, and rewards; we are not able to differentiate impact as a result of individual components. Finally, only about a third of participants had ever had sex at the 15-month follow-up resulting in small sample sizes; longer-term impact of the program once a majority of girls become sexually active is unknown.

Our analysis also has a number of strengths. The original trial was a randomized design, and thus reduces the risk of confounding biases relative to observational data. In addition, follow-up rates at both 3 and 15 months were over 95% [5].

Previous research has found that boys are more likely than girls to use mobile phones, and youth from higher socioeconomic backgrounds are also more likely to own and access mobile phones [18–20]. An evaluation of a district-wide HIV/AIDS mHealth campaign in Uganda found that women and those with lower SRH knowledge were less likely to respond to SMS quiz questions than men and those with higher SRH knowledge [9, 33]. Conversely, our findings show that girls from across socioeconomic strata and levels of SRH knowledge do actively engage in such programs when they are directly targeted, and when intervention content is tailored to their needs. For future interventions, developing such customized content is undoubtedly of critical importance, and will likely require additional qualitative work of a wider range of age groups and geographic areas. From a policy perspective, it will also be important to use additional approaches to enrol socially disadvantaged girls into the program, since many may not pursue secondary education. Actively recruiting girls from disadvantaged neighborhoods for mHealth programs, rather than promoting programs through general advertising, may be an effective way to reach these vulnerable populations.

## Conclusions

Adolescent girls in LMICs face substantial sexual and reproductive health risks, including unintended pregnancy, unsafe abortion, and HIV infection [34]. Accessing SRH information and services is challenging for adolescents due to a large number of barriers, including a lack of availability of accurate, comprehensive, and timely information [12]. As access to mobile phones among young people continues to rapidly expand, implementing mHealth programs in the area of adolescent SRH is becoming popular among academics and policymakers [5–11]. At the same time, concerns have grown that mHealth programs are not reaching vulnerable populations [18, 21, 35]. Our results suggest that mHealth platforms for adolescents have the potential to engage and increase health knowledge of adolescent girls across sociodemographic strata, including those who may be at higher risk of poor SRH outcomes.

## Appendix

**Table 4:** Text message content for Intervention and Control groups

Week	Intervention Group			Control Group
	Quiz Question/Tip text	Correct Answer	Response from SMART mHealth program	Fact text
1	SMART quiz:How many ovaries does a woman have? Reply SMT1 for 1 ovary or SMT2 for 2 ovaries. Reply to this number for free. Reply until you receive confirmation	SMT2	SMART:Right! A woman has 2 ovaries. This is where eggs are stored. She has a womb (uterus) where a fertilized egg implants and a pregnancy grows.Two fallopian tubes connect ovaries to the womb.The cervix connects the womb to the vagina. The vagina is a tube of muscle connecting cervix to outside of body	SMART fact: In 2012, malaria killed over 483,000 children under 5 years, or about 1 child every minute. Malaria kills over 45,000 adolescents per year in Africa.
2	SMART quiz:When is the most likely time that a girl can get pregnant? Reply SMT1 for days 1–7 of her menses, reply SMT2 for days 8–19, or SMT3 for days 20–28.	SMT2	SMART answer: Correct! The menstrual cycle is usually 28 days. If day 1 is the first day of your menses, then days 8–19 are the most likely time that you can get pregnant. The egg is released from the ovaries between days 8–19. If sperms are present, then the egg may be fertilized, causing pregnancy.	SMART fact:Malaria is caused by Plasmodium falciparum parasites.The only way the parasites are spread to people are thru bites of infected Anopheles mosquitoes.
3	SMART quiz: True or False: Standing up during sex can prevent a girl from getting pregnant. Reply SMT1 for true or SMT2 for false.	SMT2	SMART answer: Correct! Standing up during sex does NOT prevent pregnancy. When a man ejaculates (releases sperm), the sperms are deposited deep into the vagina immediately after ejaculation, allowing fertilization to take place. Bathing/washing will NOT prevent pregnancy either.	SMART fact:Getting malaria while pregnant is very serious. About 9% of pregnant women in Ghana die of malaria. It can also result in low birth weight babies.
Tip 1: End of week 3	SMART tip: If you have any questions about your health, you can call 0302208585 or 080028585 (Toll free- Voda only) to speak to a nurse. It is confidential.			
4	SMART:Can you be a carrier of a Sexually Transmitted Infection (STI) and NOT be aware that you have it? Reply SMT1 for yes or SMT2 for no.	SMT1	SMART:Right!You can have STI without having any symptoms or knowing you are a carrier.It can take months to see symptoms like sores, itches and problems urinating.A partner may have a STI and it may be impossible for him or you to know that he has it.Condoms or abstinence are effective ways to prevent STI	SMART fact:The first symptoms of malaria are fever, headache, and chills. These occur 2–3 days after the mosquito bite.Other symptoms are body pain and nausea.
5	SMART quiz: True or False: A woman with an untreated gonorrhea may have severe lower abdominal pains. Reply SMT1 for true or SMT2 for false.	SMT1	SMART:Right! Untreated gonorrhea may lead to severe pains in lower abdomen called pelvic inflammatory disease. It can cause infertility. It also makes it easier to get HIV. It may take months to see signs of gonorrhea in females. In males it takes days. Its important to seek treatment from a health center.	SMART:Malaria symptoms resemble diseases like pneumonia or typhoid.At health centers you can get rapid diagnostic test (just a few min) to identify the disease.
Tip 2: End of week 5	SMART Tip: Talking about reproductive health with friends, family, and a boyfriend/future boyfriend is smart. It can help you to be healthier and make good choices that are right for you. Be sure to talk to your friends about the SMART messages, and encourage them to participate! Win together!			
6	SMART quiz: True or false: A woman can wear the female condom for up to 8 h before she has sex. Reply SMT1 for true or SMT2 for false.	SMT1	SMART:Right! The female condom is made of a thin transparent and soft plastic that looks like a tube that is closed at one end.It is designed to fit into a woman’s vagina. It can be worn up to 8 h before a woman has sex.It protects against both STIs and pregnancy.It is 95% effective if worn correctly.	SMART fact: You can cure malaria with drugs called ACTs like Artesunate-Amodiaquine. ACTs combine two drugs together into each pill. They are 97% effective.
Tip 3: End of week 6	SMART Tip: Great job! Remember, if you don’t want to have sex, it’s ok to say no. Call 0302208585 or 080028585 (Toll free- Vodafone only) to speak to a nurse about strategies for saying no. It is completely confidential. You could also call this number if you have any questions bothering you.			
7	SMART:When putting on a condom, should a man unroll it all the way first before putting it on the penis? Reply SMT1 for yes or SMT2 for no.	SMT2	SMART: Right! When putting on a condom, do NOT unroll the entire condom first. Open the package, hold the tip of the condom with one hand and roll it down the penis with the other hand. Leave space at the tip to collect semen. If there is no space at the tip the condom will burst open during ejaculation.	SMART fact: The malaria parasite has developed resistance to previous drugs like chloroquine. This means the drug no longer works to cure malaria. Only ACTs cure.
8	SMART:When using a condom, when should a man pull out of the vagina after ejaculation? Reply SMT1 for while penis is still stiff or SMT2 for when penis is soft.	SMT1	SMART answer: Right! When using a condom, it is important for the man to pull his penis out right after ejaculation, while it is still stiff. If the penis gets soft then the condom could fall off inside the woman’s vagina. If this happens then it is possible that the woman will get pregnant.	SMART fact: If you take an ACT and don’t finish all the pills, the malaria parasite will survive. This builds resistance to the medicine. Always finish ACTs.
Tip 4: End of Week 8	SMART Tip: Contraception means a method to prevent pregnancy. Birth control pills and condoms are types of contraception.Condoms are only effective if you use them correctly and use them every time you have sex. Then they are 98% effective against STDs and pregnancy.Condoms do NOT cause infertility in men.			
9	SMART quiz:How often is the Pill taken (the birth control Pill)? Reply SMT1 for only after a woman has sex or reply SMT2 for once a day, everyday.	SMT2	SMART answer: Right! The Pill is taken once a day whether or not a woman has sex.If you choose to use the Pill as your contraceptive method then you must take it everyday or it is NOT effective. You can’t just take it whenever you please! It contains low and safe doses of hormones and prevents pregnancy.	SMART fact: There are no vaccines against malaria. You can prevent malaria with treated mosquito nets.Traditional medicines are not effective in curing malaria.
10	SMART quiz: True or False: Birth control pills are effective even if a woman misses taking them for 2–3 days in a row. Reply SMT1 for true or SMT2 for false.	SMT2	SMART answer: Right! The Pill is NOT effective if a woman misses it for 2 or 3 days in a row. The Pill must be taken everyday and if a woman stops taking it then she may get pregnant after 2–3 days. It does NOT take 6 months to become pregnant after stopping birth control.	SMART fact:Children who survive episodes of severe malaria may develop learning problems, brain damage, or anemia (low iron in body which affects their growth).
11	SMART:True or False:A woman should take a rest from the Pill every year because the pills build up in the body over time. Reply SMT1 for true or SMT2 for false.	SMT2	SMART answer: Right! The Pill does NOT build up in the body so women do NOT need to take a rest from the Pill. If a woman has side effects like nausea, switching to another type or brand might help. The Pill protects against pregnancy but not STIs. The Pill does not cause infertility later in life.	SMART fact: Common myths about how malaria is spread are that you can get infected from working too much in the sun or eating hot foods. These are NOT true.
12	SMART quiz: True or False. Emergency contraception must be taken within 1 h of unprotected sex. Reply SMT1 for true, and SMT2 for false.	SMT2	SMART: Right! Emergency contraception (like Postinor-2) is a method to reduce chance of pregnancy after unprotected sex or when a condom breaks. The 2 pills must be taken within 5 DAYS of unprotected sex (that’s 120 h). It should only be used for emergencies, not as a regular method of contraception.	SMART fact:Increased prevention of malaria with nets and treatment with ACTs have led to more than 3million lives saved since 2010, mostly children under 5 yrs.

**Table 5:** Knowledge Test items

Item	% responding correctly at baseline	% missing at baseline
Standing up during sex can help prevent pregnancy. (FALSE)	29	4
Condoms cause infertility in men. (FALSE)	37	4
To put on a condom, you should first unroll it all the way and then try to put it on the penis. (FALSE)	7	5
When putting on a condom, it is important to leave space at the tip. (TRUE)	28	6
When using a condom, it is important for the man to pull his penis out right after ejaculation, while it is still stiff. (TRUE)	18	5
Birth control pills (known as The Pill) are taken once every day, whether or not you have sex. (TRUE)	21	6
Birth control pills protect against sexually transmitted infections. (FALSE)	46	4
Birth control pills are effective even if a woman misses taking them for two or 3 days in a row. (FALSE)	17	5
It is important that women should “take a rest” from the pill every year because the pills build up in a woman’s body over time. (FALSE)	7	4
If a woman is having side effects with one kind of pill, switching to another type or brand might help. (TRUE)	15	7
After a woman stops taking birth control pills, she is unable to get pregnant for at least 6 months. (FALSE)	19	5
The female condom can be worn up to 8 h before having sex. (TRUE)	7	6
Emergency contraception must be taken within 1 h of having unprotected sex. (FALSE)	8	4
Symptoms of gonorrhea in females will appear the day after becoming infected. (FALSE)	33	4
Gonorrhea infection makes it easier to get HIV and other STIs and pass them to sex partners. (TRUE)	52	4
If left untreated, sexually transmitted infections like gonorrhea can cause infertility in both men and women. (TRUE)	63	3
A woman with an untreated gonorrhea may have severe lower abdominal pains. (TRUE)	50	4
If day 1 is the first day of a woman’s period, she has the greatest chance of becoming pregnant during days 8–19. (TRUE)	47	3
You can have a sexually transmitted infection without having any symptoms or knowing you are a carrier. (TRUE)	44	4
Every woman has 1 ovary where her eggs are stored. (FALSE)	30	1
STI symptoms can include sores, itches, and problems urinating. (TRUE)	Only asked at follow-up	
Postinor-2 is a type of emergency contraception. (TRUE)	Only asked at follow-up	
The female condom protects against both sexually transmitted infections and pregnancy. (TRUE)	Only asked at follow-up	
Washing/bathing oneself after sex can prevent pregnancy.(FALSE)	Only asked at follow-up	

**Table 6:** Comparison of models for factors associated with number of responses, and full results for outcomes of responded to any message and self-reported message receipt

	Total Number of Responses			Responded to any msg	Self-reported received msgs at least once/week
	Poisson (IRR)	Negative Binomial (IRR)	Linear regression	Logistic regression (OR)	Logistic regression (OR)
Sexual experience					
Not active at baseline (Ref)	1 (1,1)	1 (1,1)	0 (0,0)	1 (1,1)	1 (1,1)
Sexually active at baseline	1.08 (0.86,1.37)	1.07 (0.84,1.37)	0.68 (−1.69,3.05)	1.60 (0.49,5.22)	1.99 (0.26,15.2)
Refused	0.93 (0.59,1.48)	0.94 (0.59,1.50)	−0.55 (−4.78,3.68)	0.79 (0.13,4.93)	0.95 (0.16,5.64)
Missing	0.57 (0.28,1.19)	0.56 (0.25,1.24)	−3.44 (−7.47,0.60)	0.33 (0.041,2.75)	0.22 (0.034,1.46)
Baseline knowledge					
No knowledge deficit (Ref)	1 (1,1)	1 (1,1)	0 (0,0)	1 (1,1)	1 (1,1)
Knowledge deficit	0.92 (0.78,1.09)	0.90 (0.78,1.05)	−0.63 (−2.20,0.94)	0.95 (0.41,2.21)	0.58 (0.21,1.55)
Parental education					
Both parents low	1.22^*^ (1.03,1.46)	1.23^*^ (1.04,1.47)	1.78 (−0.064,3.63)	1.98 (0.28,14.1)	0.79 (0.18,3.41)
One parent high	0.93 (0.84,1.03)	0.95 (0.84,1.07)	−0.55 (−1.45,0.34)	0.83 (0.30,2.31)	1.13 (0.29,4.50)
Both parents high (Ref)	1 (1,1)	1 (1,1)	0 (0,0)	1 (1,1)	1 (1,1)
Other/Missing	1.02 (0.82,1.26)	1.02 (0.82,1.27)	0.14 (−1.90,2.19)	0.55 (0.23,1.35)	0.75 (0.20,2.85)
Parental support					
Low	1.04 (0.85,1.28)	1.04 (0.83,1.31)	0.31 (−1.62,2.23)	1.71 (0.70,4.19)	1.60 (0.69,3.67)
High (Ref)	1 (1,1)	1 (1,1)	0 (0,0)	1 (1,1)	1 (1,1)
Home economics class in school	1.14 (0.97,1.33)	1.13 (0.94,1.35)	1.01 (−0.47,2.49)	1.35 (0.98,1.87)	1.46 (0.40,5.35)
School Category					
Private	1 (1,1)	1 (1,1)	0 (0,0)	1 (1,1)	1 (1,1)
High Quality	1.00 (0.86,1.15)	0.95 (0.78,1.17)	−0.037 (−1.46,1.38)	0.73 (0.41,1.31)	1.49 (0.42,5.23)
Low Quality	0.87 (0.73,1.04)	0.83^*^ (0.69,1.00)	−1.11 (−2.77,0.55)	0.62 (0.36,1.07)	0.89 (0.29,2.73)
Observations	204	204	204	204	191

Note: Parantheses show 95% confidence intervals. 1 observation had missing value for parental support and was dropped from analysis. *Ref* reference category, *IRR* incidence rate ratios, *OR* Odds ratios, *msgs* messages

**Table 7:** Linear regression results for impact of mHealth treatment on sexual and reproductive health knowledge z-score, stratified by target subgroups, at 3-month and 15-month follow-ups

	Parental education				Baseline knowledge		Sexually active at baseline			Parental Support	
	Both parents low	One parent high	Both parents high	Missing/Other	No knowledge deficit	Knowledge deficit	Not sexually active	Sexually active	Refused	Low	High
Panel A: 3-month follow-up											
Treatment	2.148***	1.679***	1.481***	1.982***	1.388***	1.700***	1.722***	1.836***	1.661***	1.748***	1.477***
	(0.301)	(0.271)	(0.262)	(0.235)	(0.132)	(0.257)	(0.215)	(0.332)	(0.364)	(0.186)	(0.310)
Home econ. Class	0.446	0.461**	0.482**	0.903**	0.590*	0.634**	0.772***	−0.353	0.661	0.718***	0.440
	(0.747)	(0.217)	(0.219)	(0.390)	(0.301)	(0.226)	(0.182)	(0.586)	(0.412)	(0.174)	(0.285)
High Quality public school	0.989	−0.286	0.384	−0.337	0.285	−0.0810	−0.0568	0.563	0.0339	0.165	−0.0607
	(0.682)	(0.331)	(0.309)	(0.280)	(0.238)	(0.276)	(0.228)	(0.600)	(0.279)	(0.208)	(0.352)
Low Quality public school	0.876	0.00474	0.250	−0.854***	0.223	−0.217	−0.0882	0.448	−0.166	−0.000159	0.0725
	(0.616)	(0.208)	(0.329)	(0.224)	(0.214)	(0.210)	(0.171)	(0.676)	(0.265)	(0.195)	(0.268)
Constant	−1.055**	0.0161	−0.394*	−0.281	0.0442	−0.664***	−0.459**	0.468	−0.348	−0.538***	−0.0570
	(0.441)	(0.141)	(0.199)	(0.495)	(0.255)	(0.168)	(0.198)	(0.533)	(0.408)	(0.154)	(0.306)
Observations	37	108	236	97	242	236	336	62	61	324	153
R-squared	0.647	0.359	0.307	0.498	0.339	0.411	0.401	0.415	0.369	0.416	0.263
p-value_interaction_	0.12				0.10		0.78			0.15	
Panel B: 15-month follow-up											
Treatment	0.881***	0.812***	0.832***	1.181***	0.923***	0.598***	0.829***	0.980**	0.929**	0.887***	0.895***
	(0.305)	(0.191)	(0.246)	(0.173)	(0.116)	(0.159)	(0.127)	(0.410)	(0.433)	(0.157)	(0.171)
Home econ. Class	0.616	−0.0862	−0.0355	0.277	0.106	−0.0325	0.0843	−1.189*	0.810*	0.0363	0.159
	(0.784)	(0.286)	(0.252)	(0.306)	(0.263)	(0.245)	(0.235)	(0.602)	(0.447)	(0.219)	(0.268)
High Quality public school	1.475***	0.246	−0.0608	0.0939	0.284	0.139	0.274	0.673	−0.235	0.261	−0.0683
	(0.190)	(0.461)	(0.341)	(0.214)	(0.235)	(0.277)	(0.253)	(0.577)	(0.392)	(0.236)	(0.300)
Low Quality public school	0.950***	0.449	0.139	−0.329	0.369	0.0488	0.351	0.704	−0.355	0.327	0.0116
	(0.240)	(0.487)	(0.352)	(0.217)	(0.231)	(0.266)	(0.249)	(0.651)	(0.332)	(0.238)	(0.297)
Constant	−0.651	0.676***	0.753**	0.442	0.669***	0.503	0.382	1.646***	0.0955	0.446*	0.678***
	(0.708)	(0.217)	(0.281)	(0.396)	(0.225)	(0.302)	(0.282)	(0.489)	(0.409)	(0.256)	(0.231)
Observations	36	113	225	100	241	233	345	65	63	326	147
R-squared	0.434	0.138	0.111	0.277	0.210	0.077	0.146	0.282	0.193	0.149	0.144
p-value_interaction_	0.51				0.11		0.37			0.99	

*Notes*: Each column is separate regression model, stratified by variable in column header. Clustered standard errors at school level in parentheses. *** *p* < 0.01, ** *p* < 0.05, * *p* < 0.1. The p-value_interaction_ is from linear hypothesis test of equality of coefficients for treatment variable, calculated after model interacting treatment with indicator for subgroup. Home econ. = Home economics class in school. Reference category for category of schools is Private school

## Abbreviations

CI: Confidence interval
IRR: Incidence rate ratio
LMIC: Low- and middle-income country
mHealth: Mobile health
OR: Odds ratio
SRH: Sexual and reproductive health
STI: Sexually transmitted infection
